# Enhanced Pyroelectric Performance of Lead-Free Zn-Doped Na_1/2_Bi_1/2_TiO_3_-BaTiO_3_ Ceramics

**DOI:** 10.3390/ma15010087

**Published:** 2021-12-23

**Authors:** Satyanarayan Patel, Kodumudi Venkataraman Lalitha, Nishchay Saurabh

**Affiliations:** 1 Department of Mechanical Engineering, Indian Institute of Technology Indore, Khandwa Road Simrol, Indore 453552, Madhya Pradesh, India; phd2001203003@iiti.ac.in; 2 Institute of Materials Science, Technische Universität Darmstadt, 64287 Darmstadt, Germany

**Keywords:** pyroelectric, lead-free ceramics, dielectric, NBT-BT

## Abstract

Lead-free Na_1/2_Bi_1/2_TiO_3_-BaTiO_3_ (NBT-BT) has gained revived interest due to its exceptionally good high power properties in comparison to commercial lead-based piezoelectrics. Recently, Zn-modified NBT-BT-based materials as solid solution and composites have been reported to exhibit enhanced depolarization temperatures and a high mechanical quality factor. In this work, the pyroelectric properties of Zn-doped NBT-6mole%BT and NBT-9mole%BT ceramics are investigated. The doped compositions of NBT-6BT and NBT-9BT feature a relatively stable pyroelectric property in a wide temperature range of ~37 K (300–330 K) and 80 K (300–380 K), respectively. A threefold increase in detector figure of merit is noted for 0.01 mole Zn-doped NBT-6mole% BT at room temperature in comparison to undoped NBT-6mole%BT and this increase is higher than those of major lead-free materials. A broad range of the temperature-independent behavior for the figures of merit was noted (303–380 K) for Zn-doped NBT-6mole% BT, which is 30 K higher than the undoped material. The large pyroelectric figures of merit and good temperature stability renders Zn-doped NBT-BT an ideal candidate for pyroelectric detector and energy harvesting applications.

## 1. Introduction

Pyroelectric materials have been widely investigated for various industrial applications, such as infrared detectors, thermal cameras, sensors, medical devices, etc. [1,2,3]. In addition to these applications, waste thermal energy conversion for low-power electronic devices and battery-less wireless sensor applications receive significant industrial and academic interest [4]. In this direction, mostly Pb[Zr_(x)_Ti_(1-x)_]O_3_ (PZT), Pb(Mg_1/3_Nb_2/3_)O_3_-PbTiO_3_ and their solid solutions are studied due to their impressive pyroelectric, piezoelectric and dielectric properties [5,6,7]. However, the toxicity of lead oxide limits their use in electronic devices (European Union and many other countries) and demands alternative lead-free materials [2,8]. Therefore, various lead-free ferroelectric ceramics have been developed and documented in the literature for the past few decades, that demonstrate excellent pyroelectric properties [9,10]. Among the investigated lead-free materials, Na_1/2_Bi_1/2_TiO_3_ and its solid solution are promising because of their ease of fabrication. K_0.5_Na_0.5_NbO_3_ (KNN) and Ba_0.85_Ca_0.15_Zr_0.1_Ti_0.9_O_3_ (BCZT) also exhibit promising piezoelectric and ferroelectric properties; however, fabrication of KNN is difficult compared to NBT-based compositions [1,11]. Na_1/2_Bi_1/2_TiO_3_-xBaTiO_3_ (NBT-BT) has been studied by various researchers, at 6–7 mole% BT, wherein it exhibits a morphotropic phase boundary (MPB) and features high remanent polarization, large piezoelectric constant (d_33_) and low coercive field [12,13]. Furthermore, at the MPB, poling is much easier due to the low coercive field, favorable for pyroelectric applications [14]. NBT-xBT phase diagram is complex and presents various phase states/transitions, such as ferroelectric (FE), non-polar (non-ergodic relaxor) and paraelectric (PE) rendering it beneficial, as one can utilize the large pyroelectric currents at the vicinity of these phase transitions [15,16].

0.94Na_1/2_Bi_1/2_TiO_3_-0.06BaTiO_3_ (NBT-6BT) has been extensively investigated from the ferroelectric, dielectric and piezoelectric application points of view; however, there are few publications on pyroelectric properties [17,18,19,20]. Takenaka et al. have reported a high pyroelectric coefficient (*p*) = 3.9 × 10^−4^ C m^−2^ K^−1^ for NBT-6BT [21]. In another study, Felix et al. reported pyroelectric behavior of NBT-xBT (4 ≤ x ≤ 6 mole%) and demonstrated that NBT-6BT exhibits higher *p* compared to other compositions (attributed to MPB) and features a similar *p* akin to PZT [22]. Guo et al. have studied Zr-doped NBT-xBT (0% ≤ x ≤ 12%) ceramics and found that *p* varies between 5.7 × 10^−4^ and 22.1 × 10^−4^ C m^−2^ K^−1^ from room temperature to depolarization temperature (T_d_) [10]. Abe et al. investigated MnO_2_-doped NBT-6BT and reported *p* = 3.5 × 10^−4^ C m^−2^ K^−1^ at room temperature [23]. Recently, Balakat et al. have been extensively investigating the pyroelectric properties of 0.94Na_x_Bi_y_TiO_3_-0.06Ba_z_TiO_3_ [24], 0.94Na_1/2_Bi_1/2_TiO_3_-0.06Ba_1+x_TiO_3_ [25], La-doped NBT-6BT [26], Ta-doped NBT-6BT [27], and La, Ta-doped NBT-6BT [28]. The depolarization temperature (T_d_) is lowered using these doping strategies; however, only a small improvement was observed in the pyroelectric figures of merit (FOMs). Here, T_d_ plays an important role from the practical application point of view, since the pyroelectric and piezoelectric properties vanish above this temperature. Lalitha et al. reported on Zn-doped NBT-6BT and NBT-9BT, that featured an increase in T_d_ to 423 K and 469 K, respectively [29]. Saurabh N. et al. used nonstoichiometric 0.94Na_x_Bi_y_TiO_3_-0.06BaTiO_3_ to analyze the effect on T_d_ and reported an enhanced pyroelectric coefficient of 105 × 10^−4^ C m^−2^ K^−1^ for the nonstoichiometric sample at room temperature [30]. Further, Li et al. used the composite approach with nonstoichiometric (1-x)0.94Na_0.48_Bi_0.44_TiO_3_-0.06BaTiO_3_ and ZnO to enhance the pyroelectric properties and T_d_ [31].

In order to assess the performance of pyroelectric materials, a variety of FOMs have been developed for different applications [32,33,34]. These include FOMs for current responsivity ($F_{i}$), detectivity ($F_{d}$), voltage responsivity ($F_{v}$), the pyroelectric figure of merit ($F_{c}$) and energy harvesting ($F_{e}$ and $F_{e}^{*}$). Therefore, to derive the best performance out of pyroelectric materials, not only a higher pyroelectric coefficient (*p*) but also small relative dielectric permittivity ($\varepsilon_{r}$) and loss (tanδ) are desirable [6]. Therefore, an optimum tradeoff between *p*, $\varepsilon_{r}$, tanδ and specific heat capacity (*C*) is required to enhance FOMs. In addition to this, materials should demonstrate temperature-independent pyroelectric performance. Hence, several methods are used to improve the FOMs, such as composite fabrication, porous ceramics and chemical modification [2,35,36,37]. However, researchers continue to explore new materials for efficient usage. In the present work, Zn-doped NBT-6BT and NBT-9BT ceramics are explored to characterize the pyroelectric performance. Hence, a comprehensive set of dielectric, pyroelectric and piezoelectric characterization is undertaken, along with the estimation of relevant FOMs. This work suggests an avenue to design pyroelectric devices with temperature-independent performance in a wide temperature range, possibly substituting lead-based ceramics.

## 2. Materials and Methods

0.94Na_1/2_Bi_1/2_TiO_3_-0.06BaTiO_3_ (NBT-6BT) and 0.91Na_1/2_Bi_1/2_TiO_3_-0.09BaTiO_3_ powders doped with xZnO (x = 0, 0.005, 0.01; x denotes number of moles) were prepared by a solid-state synthesis route. The raw powders of Bi_2_O_3_ (99.975%), BaCO_3_ (99.8%), Na_2_CO_3_ (99.5%), TiO_2_ (99.6%) and ZnO (99.99%) (all Alfa Aesar) were weighed according to their stoichiometric ratio. Using a planetary ball mill, the raw materials were ball-milled for 12 h in ethanol at 250 rpm (Fritsch Pulverisette 5). The resultant slurries were dried and calcined at 900 °C for 3 h in closed alumina crucibles with a heating rate of 5 K min^−1^. The calcined powders were remilled again for 6 h in ethanol at 250 rpm. Sintering was done at 1100 °C for 1 h at a heating rate of 5 K min^−1^. In addition, to avoid volatilization of Na^+^ and Bi^3+^ during the sintering process, samples were embedded in a powder bed of the same composition. The relative density of the samples was measured using the Archimedes method. A polished and thermally etched (100–150 °C below the sintering temperature for 15 min) sample was used for scanning electron microscopy (SEM) (Philips XL30 FEG). For the electrical characterization, samples were ground to a thickness of 0.6 mm and sputtered with silver electrodes. Samples were poled in silicone oil by applying a DC field 6 kV mm^−1^ at room temperature for 15 min and the piezoelectric, dielectric and pyroelectric measurements were performed after 24 h. The piezoelectric constant (d_33_) was measured using a d_33_ meter (PM 300; Piezo Test) under a load of 2 N at room temperature. An HP analyzer was used to measure the temperature and frequency-dependent permittivity using a programmable furnace (ramp rate of 2 K min^−1^). The pyroelectric current (*I*) was measured in a short circuit mode, utilizing a constant heating rate of 3 K min^−1^, while the discharge currents were monitored by an electrometer (Model 617; Keithley). The measurements were performed in two different methods. The samples were measured in one complete heating (above T_d_) and cooling cycle in one set of measurements. In another set of experiments, the samples were subjected to temperature cycling (heating at 3 K min^−1^ and natural cooling) up to ~20 K below the T_d_ (10 cycles). The pyroelectric current was measured in the whole temperature range (above T_d_) after the thermal cycling. The measured *I* is used for the estimation of the pyroelectric coefficient (*p*) and the FOMs.

## 3. Results and Discussion

Phase purity analysis using XRD has been previously reported by Lalitha et al. [29] and samples from the same batch have been used in the present work. Hence, XRD data are not presented here. The X-ray diffraction pattern of NBT-yBT-xZn (y = 6, 9 and x = 0.005 and 0.01) ceramics correspond to the perovskite pattern. Secondary phases were not detected within the sensitivity of XRD, following previous reports [29]. This confirms the complete incorporation of ZnO into the NBT-6BT and NBT-9BT host lattice.

**Figure 1:**
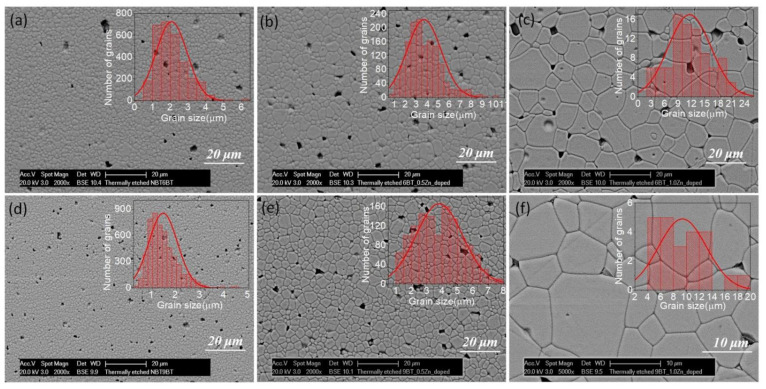
Scanning electron micrographs of thermally etched and polished surface of (**a**) NBT-6BT, (**b**) NBT-6BT-0.005Zn, (**c**) NBT-6BT-0.01Zn, (**d**) NBT-9BT, (**e**) NBT-9BT-0.005Zn and (**f**) NBT-9BT-0.01Zn. The inset shows the corresponding grain size distribution.

**Figure 2:**
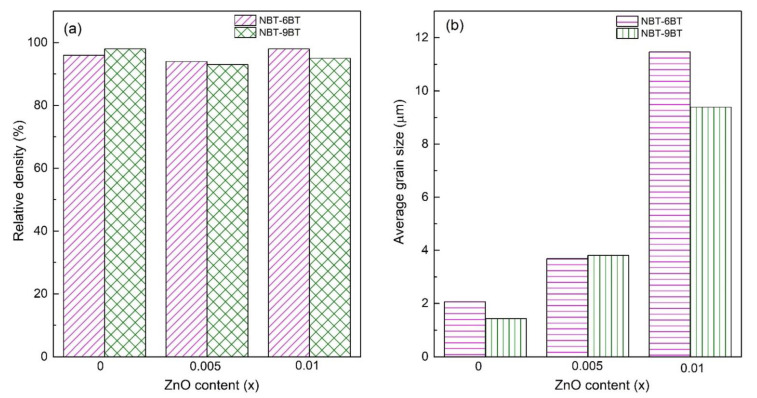
(**a**) Relative density and (**b**) average grain size as a function of Zn content for NBT-yBT-xZn (y = 6, 9 and x = 0, 0.005, 0.01) ceramics.

Figure 1 features the surface morphology of the NBT-yBT-xZn (y = 6, 9 and x = 0.005 and 0.01) ceramics and the insets present the corresponding grain size distribution. The micrographs feature a dense microstructure and the grain size increases with increasing ZnO content. Moreover, compositional segregation is not observed in the SEM images. Figure 2a shows the relative density as a function of ZnO content, which is in the range of 93–98% for NBT-yBT-xZn (y = 6, 9 and x = 0.005 and 0.01) ceramics. The relative density for 0.005 mole ZnO added NBT-6BT, i.e., 93% increases to 98% for 0.01 mole of ZnO. Further, the average grain size as a function of ZnO content was estimated and is shown in Figure 2b. It is observed that with increasing ZnO content, the average grain size increases from ~2–11.4 µm and 1.4–9.3 µm for NBT-6BT and NBT-9BT, respectively.

**Figure 3:**
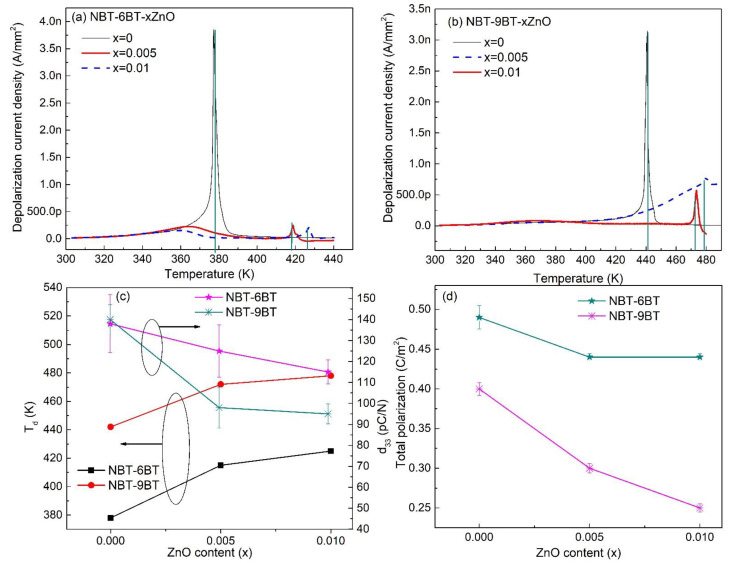
Depolarization current density after one complete cycle as a function of temperature for (**a**) NBT-6BT-xZn (x = 0, 0.005, 0.01) and (**b**) NBT-9BT-xZn (x = 0, 0.005, 0.01). The vertical green lines denote the T_d_. (**c**) Depolarization temperature (T_d_) and piezoelectric coefficient (d_33_) of NBT-yBT (y = 6, 9) as a function of ZnO content. (**d**) Total polarization of NBT-yBT (y = 6, 9) as a function of ZnO content.

The depolarization temperature (T_d_) obtained from the depolarization current density (I_A_) for NBT-6BT and NBT-9BT is shown in Figure 3a,b. T_d_ is the temperature at which the macroscopic polarization of the material disappears and can be observed with a sharp increment in the temperature-induced I_A_. NBT-6BT and NBT-9BT exhibit T_d_ of 377 K and 442 K, respectively. With Zn-doping in NBT-6BT and NBT-9BT, T_d_ increases to 415 K, 425 K and 472 K, 478 K, respectively. The graphical variation of T_d_ with ZnO content is shown in Figure 3c.

This increase in the depolarization temperature of NBT-yBT-xZn (y = 6, 9 and x = 0, 0.005, 0.01) has been attributed to the large ionic polarizability of the Zn^2+^ [38] and onset of tetragonal distortion, stabilizing the ferroelectric domain structure [29]. Additionally, the increase in the grain size could also stabilize the ferroelectric state, leading to higher T_d_ [39]. Piezoelectric constant (d_33_) for NBT-6BT is observed to decrease from 137 to 124 pC/N and 114 pC/N with increasing Zn concentration to 0.005 and 0.01, respectively, as seen in Figure 3c. A similar decrease in d_33_ is observed for NBT-9BT, where it decreased by 37% and 32%, with increasing ZnO content. The decrease in d_33_ may be due to the hardening caused by Zn^2+^ doping at the B site of NBT-yBT [29]. Figure 3d depicts the total polarization of NBT-6BT and NBT-9BT, which decreases with the increase in Zn concentration. Further, d_33_ and polarization also decreases with an increase in grain size, due to acceptor (Zn)-doping effects. Hence, the minimum d_33_ and polarization was observed for 0.01 mole ZnO doped NBT-6BT and NBT-9BT, featuring larger grain size. A comprehensive discussion on the effect of Zn-doping on the polarization and piezoelectric properties is discussed by Lalitha et al. [29].

**Figure 4:**
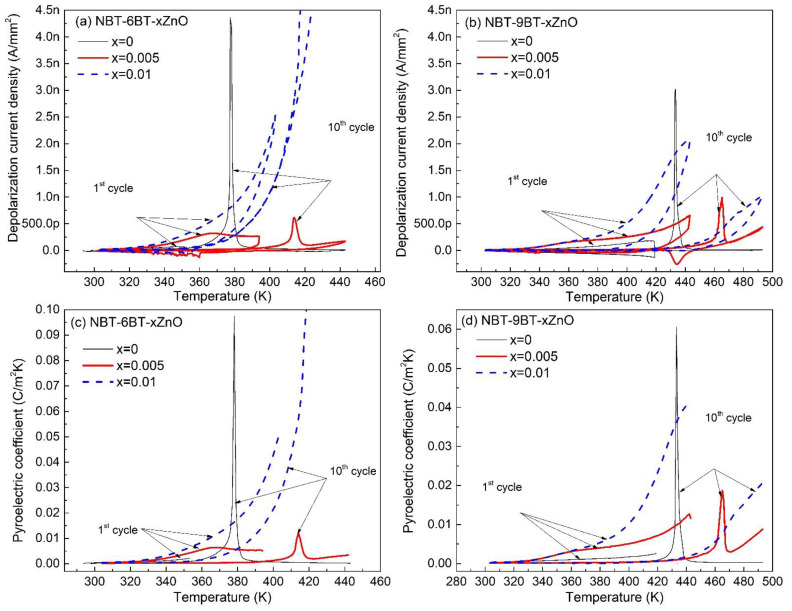
Depolarization current density and pyroelectric coefficient (*p*) for 1st and after 10th cycle as a function of temperature for (**a**), (**c**) NBT-6BT-xZn (x = 0, 0.005, 0.01) and (**b**), (**d**) NBT-9BT-xZn (x = 0, 0.005, 0.01), respectively.

The pyroelectric coefficient can be measured by various methods, including direct or indirect methods. However, in the present study, a direct measurement of a pyroelectric coefficient is used, owing to its higher accuracy. The pyroelectric coefficient is estimated by the Byer–Roundy technique based on the following equation [40]:

(1) $$I_{p}=A\times p\times \frac{dT}{dt}$$

where *I_p_* is the depolarization current, *A* is the area of the electrodes, *p* is the pyroelectric coefficient and dT/dt is the heating or the cooling rate. Figure 4a,b shows the depolarization current density (i.e., depolarization current per unit area of electrodes, I_A_) after the 1st cycle (i.e., below the T_d_) and after the 10th complete heating and cooling cycle (above T_d_), for NBT-6BT and NBT-9BT, respectively. This has been ascertained to minimize the effect of trapping and de-trapping charge arising from heating. These trapped charges may give an erroneous value to the depolarization current and hence, affecting the pyroelectric constant. Thus, the combined effect of the multiple heating and natural cooling cycles (i.e., ten in the present study) reduces any charge trapping effect. In addition, in practical application, multiple heating–cooling cycles are employed. It is important to note that, to avoid thermal depolarization, the samples were heated up to 20 K below T_d_ during multiple heating cycles. Finally, after the tenth cycle, the samples were heated above the T_d_. The variations in I_A_ for the first and after the tenth cycle for NBT-yBT-xZn (y = 6, 9 and x = 0, 0.5, 1) are shown in Figure 4a,b.

The pyroelectric coefficient *p*, calculated using Equation (1) for NBT-6BT-xZn and NBT-9BT-xZn, is shown in Figure 4c,d, respectively. *p* at room temperature for NBT-6BT and NBT-9BT is calculated to be 3.40 × 10^−4^ C m^−2^ K^−1^ and 1.72 × 10^−4^ C m^−2^ K^−1^, respectively. However, doping with ZnO in NBT-6BT and NBT-9BT did not significantly alter the *p* at room temperature. The maximum *p* is noted for NBT-6BT and NBT-9BT as 974.8 × 10^−4^ C m^−2^ K^−1^ (at 378 K) and 605 × 10^−4^ C m^−2^ K^−1^ (at 432 K), respectively. Moreover, the maximum *p* could not be increased by Zn-doping, but a temperature-independent behavior of *p* is observed. A temperature-independent range of 300–330 K and 300–380 K for *p* is noted for NBT-6BT and NBT-9BT, respectively. These values are increased to 370 K and 340 K for NBT-6BT and 380 K and 395 K for NBT-9BT with 0.005 and 0.01 moles of ZnO addition. Hence, NBT-yBT-xZn (y = 6, 9 and x = 0, 0.005, 0.01) with higher T_d_ can be used in devices for electronic sensing or energy harvesting, as most of the devices heat up during their operation nearly in the range of 333 K–363 K. In addition, the increase in T_d_ is highly relevant for energy harvesting applications, where a larger temperature difference is demanded. However, these compositions can also be useful to study the temperature-independent FOM. Thus, to understand the FOMs, it is essential to analyze the dielectric constant and loss, as the FOMs are largely dependent on the *p* and dielectric constant and loss.

**Figure 5:**
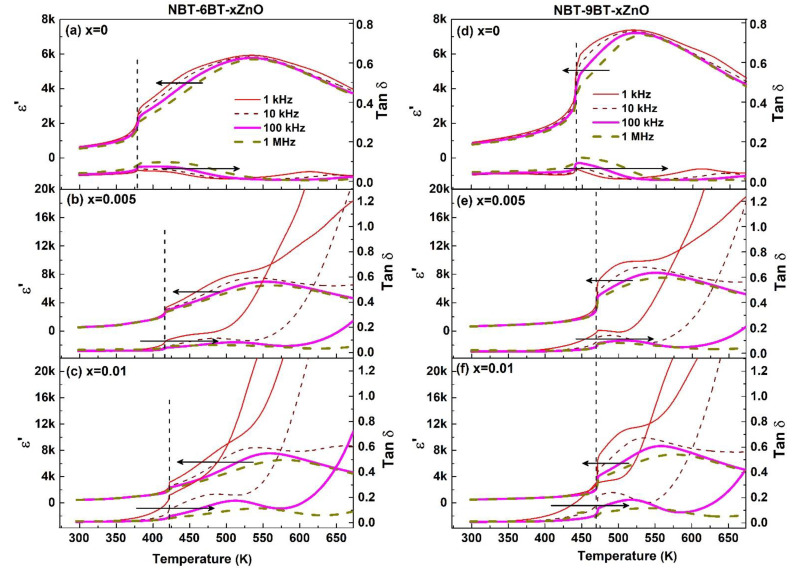
Dielectric permittivity ($\varepsilon_{r}$) and loss (tanδ) of poled samples as a function of temperature at various frequencies for (**a**) NBT-6BT, (**b**) NBT-6BT-0.005Zn, (**c**) NBT-6BT-0.01Zn, (**d**) NBT-9BT, (**e**) NBT-9BT-0.005Zn, and (**f**) NBT-9BT-0.01Zn.

The dielectric properties associated with the compositions can be observed by the variation in the dielectric permittivity ($\varepsilon_{r}$) and loss (tanδ) as a function of temperature as well as frequency is shown in Figure 5. The details of non-ergodic relaxor state (doped and undoped NBT-6BT), spontaneous ferroelectric order at room temperature (doped NBT-9BT) and ergodic state at high temperature have been discussed in detail in previous work [29]. Figure 5 shows that two dielectric anomalies are present in the poled state, owing to different transition processes. The first anomaly in the graph of dielectric constant is the ferroelectric to relaxor transition temperature (T_F-R_) and the second anomaly T_m_ shows the transition from relaxor to paraelectric. T_F-R_ is comparable to T_d_ with minor changes (~2–3 K) noted by some other research groups, in the absence of any other external stimuli (strong electric field or stress). Figure 5a shows the T_F-R_ to be 376 K for NBT-6BT. An increased T_F-R_, i.e., 413 K and 423 K with 0.005 and 0.01 moles of ZnO, is obtained in NBT-6BT (Figure 5b,c). A similar trend is seen for NBT-9BT, where T_F-R_ is observed to be 445 K, which increases to 470 K with Zn-doping, as seen in Figure 5d–f.

The increment in T_F-R_ with increasing Zn concentration is attributed to stabilizing the ferroelectric order at a higher temperature [29]. $\varepsilon_{r}$ at room temperature for NBT-6BT and NBT-9BT is 678 and 912 at 1 kHz. However, with the addition of 0.005 and 0.01 mole of ZnO, $\varepsilon_{r}$ decreases to 561, 468 for NBT-6BT and 691 and 520 for NBT-9BT at 1 kHz, respectively. A decrease in tanδ is observed with increasing Zn concentration at room temperature. This decrease in dielectric constant and loss is due to the acceptor-doping effect of Zn in NBT-yBT [29]. Furthermore, the dielectric constant and loss at room temperature decreases with increase in grain size. The lower dielectric constant can be a result of lower domain wall density (corresponding to larger grain size). The dielectric constant at T_F-R_ is decreased by 23% (NBT-9BT-0.005Zn) and 45% (NBT-9BT-0.01Zn) compared to NBT-9BT; however, for NBT-6BT, it remains almost constant. This decrease in the dielectric constant at room temperature and increase in T_F-R_ can be beneficial to enhance the FOM. However, $\varepsilon_{r}$ and tanδ for Zn-doped materials increases rapidly after T_F-R_ in the low frequency region. This rapid increase is due to enhanced conductivity in the doped compositions [29].

**Figure 6:**
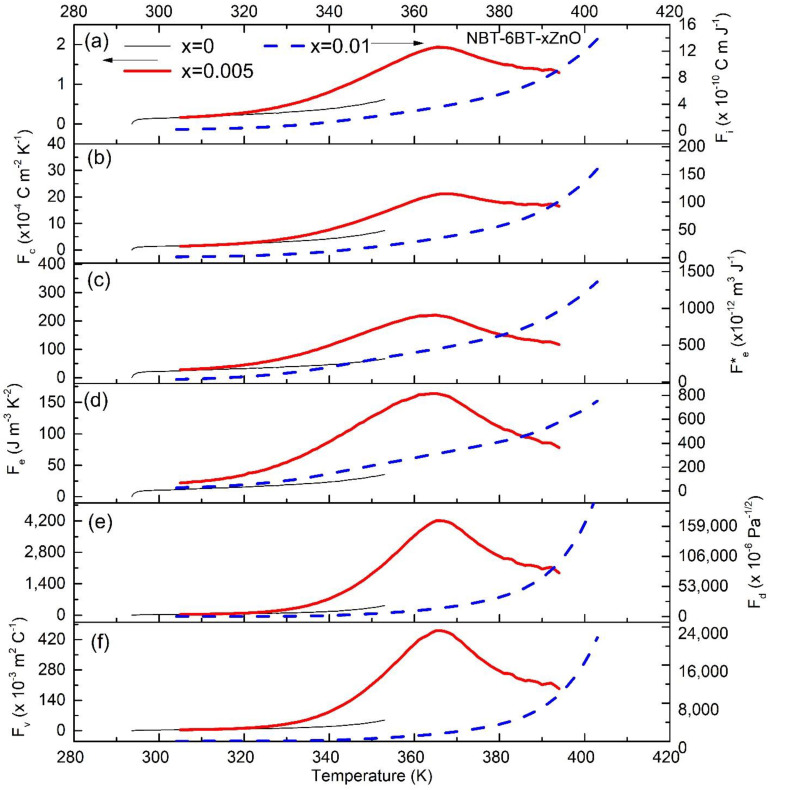
Pyroelectric figures of merit versus temperature plots of NBT-6BT-xZn (x = 0.005 and 0.01) for (**a**) current responsivity $F_{i}$, (**b**) the pyroelectric figure of merit $F_{c}$, energy harvesting (**c**) $F_{e}^{*}$ and (**d**) $F_{e}$. (**e**) detectivity $F_{d}$ and (**f**) voltage responsivity $F_{v}$.

**Figure 7:**
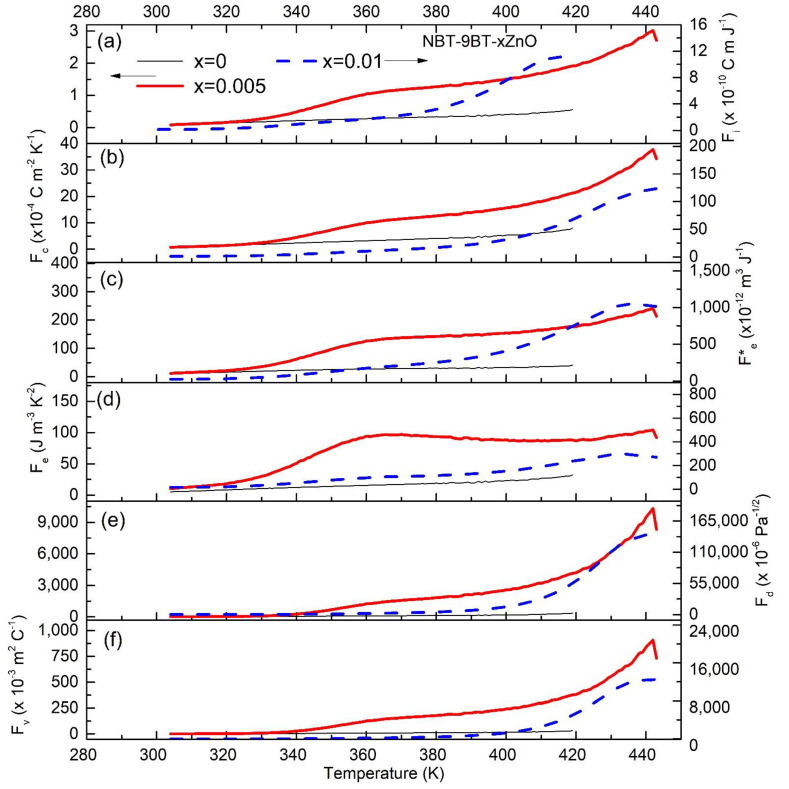
Pyroelectric figures of merit versus temperature plots of NBT-9BT-xZn (x = 0.005 and 0.01) for high (**a**) current responsivity $F_{i}$, (**b**) the pyroelectric figure of merit $F_{c}$, energy harvesting (**c**) $F_{e}^{*}$ and (**d**) $F_{e}$, (**e**) detectivity $F_{d}$ and (**f**) the voltage responsivity $F_{v}$.

**Figure A1:**
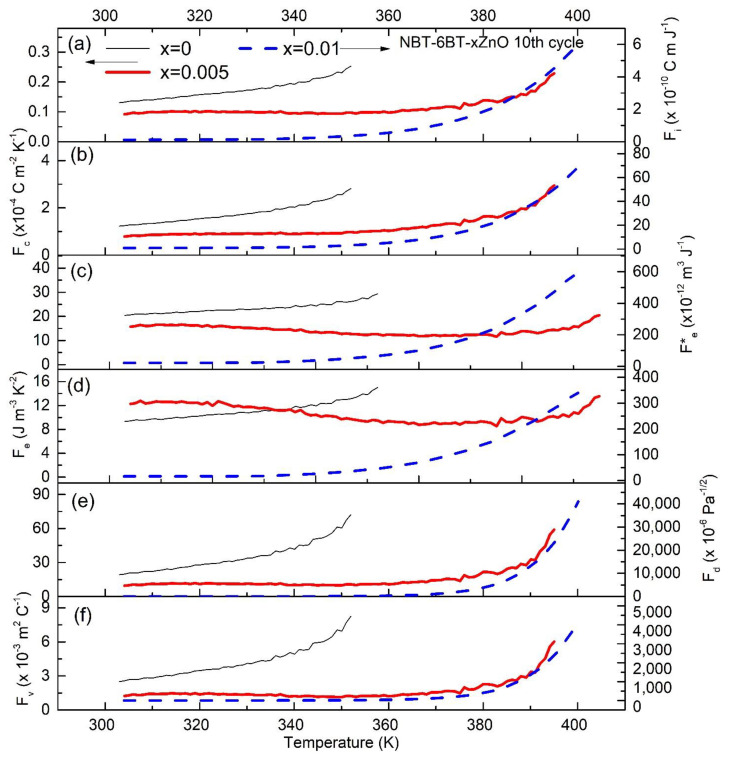
Pyroelectric figures of merit versus temperature plots after tenth heating cycle for NBT-6BT-xZn (x = 0.5 and 1%): (**a**) current responsivity ($F_{i}$), (**b**) the pyroelectric figure of merit ($F_{c}$), energy harvesting (**c**) $F_{e}^{*}$, and (**d**) $F_{e}$. (**e**) Detectivity ($F_{d}$) and (**f**) the voltage responsivity ($F_{v}$).

**Figure A2:**
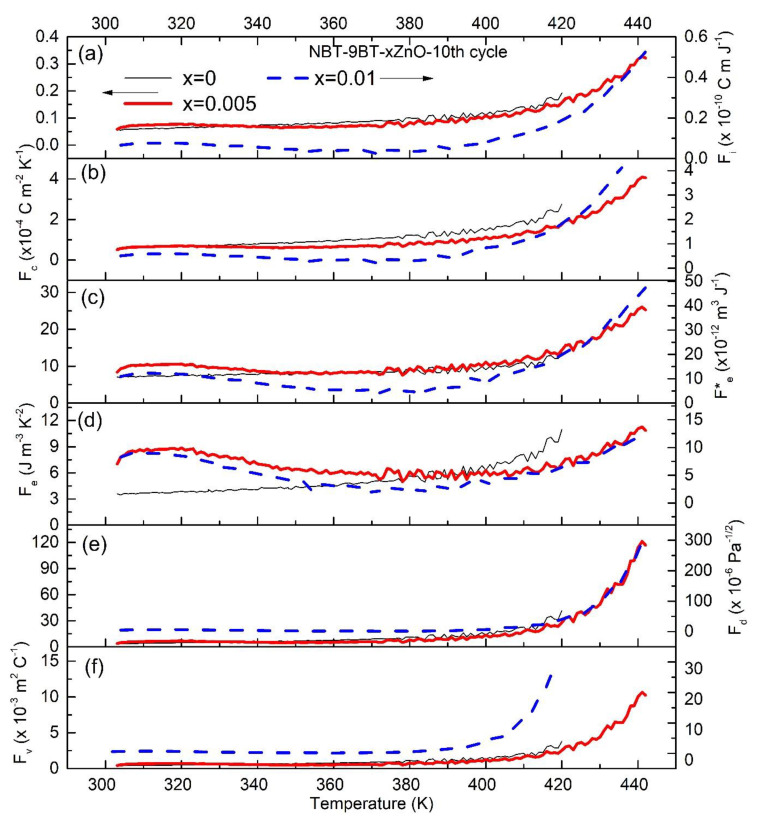
Pyroelectric figures of merit versus temperature plots after tenth heating cycle of NBT-9BT-xZn (x = 0.5 and 1%): (**a**) current responsivity ($F_{i}$), (**b**) the pyroelectric figure of merit ($F_{c}$), energy harvesting (**c**) $F_{e}^{*}$, and (**d**) $F_{e}$. (**e**) Detectivity ($F_{d}$) and (**f**) the voltage responsivity ($F_{v}$).

In order to further evaluate the pyroelectric performance, various pyroelectric FOMs are estimated. Therefore, *p*, $\varepsilon_{r}$ and tanδ are used to determine the various FOMs presented in Figure 6 and Figure 7 for NBT-6BT-xZn and NBT-9BT-xZn (x = 0.005 and 0.01), respectively for the first cycle of heating. The corresponding FOMs after the tenth heating cycle are shown in Figure A1 and Figure A2 (Appendix A). For high current responsivity, the relevant FOM is $F_{i}=\frac{p}{c_{v}}$, where $c_{v}$ is volume-specific heat [41]. Temperature-dependent specific heat capacity is taken from literature and $c_{v}$ is estimated as $c_{v}=C.\rho$, where ρ is the density of the material [42]. The sample densities were measured using the Archimedes principle and the obtained values are used for calculations. The pyroelectric FOM is expressed as $\left(F_{C}\right)=\frac{p}{\sqrt{\epsilon_{r}}}$. Energy harvesting FOMs are ($F_{e})=\frac{p^{2}}{\varepsilon_{r}\varepsilon_{0}}$ and ($F_{e}^{*})=\frac{p^{2}}{\varepsilon_{r}\varepsilon_{0}c_{v}^{2}}$ [4]. High detectivity based FOM is $(F_{d})=\frac{p}{c_{v}\sqrt{\varepsilon_{r}\varepsilon_{0}tan\delta }}$. Moreover, for the high voltage responsivity, the FOM is given as $(F_{v})=\frac{p}{c_{v}\;\varepsilon_{r}\varepsilon_{0}}$, where $\varepsilon_{0}$ is the permittivity of free space [4]. Figure 6 and Figure 7 show the calculated FOMs in the temperature range of 300 K–400 K and 300 K–440 K, respectively. Figure 6a and Figure 7a show that, at room temperature, $F_{i}$ increases with increasing ZnO content, which is found as 1.07, 1.41, 1.44 × 10^−10^ C m J^−1^ and 0.81, 0.73 and 1.07 × 10^−10^ C m J^−1^ for NBT-6BT-xZn and NBT-9BT-xZn (x = 0, 0.005 and 0.01).

Similarly, at room temperature, $F_{v}$ increases with increasing ZnO content, which is found as 17.9, 28.20 and 34.5 × 10^−3^ m^2^ C^−1^ and 10, 13.6 and 23 × 10^−3^ m^2^ C^−1^ for NBT-6BT-xZn and NBT-9BT-xZn (x = 0, 0.005 and 0.01), respectively, as seen in Figure 6b and Figure 7b. A similar trend is observed for pyroelectric energy harvesting FOMs ($F_{e}^{*})$ and ($F_{e})$ as seen in Figure 6c,d and Figure 7c,d.

The maximum value of FOMs ($F_{e}^{*}$) and ($F_{e}$) obtained are 1033 × 10^−12^ m^3^ J^−1^ and 281 J m^−3^ K^−2^ for NBT-9BT-0.01Zn, 1363 × 10^−12^ m^3^ J^−1^ and 753 J m^−3^ K^−2^ for NBT-6BT-0.01Zn, which are much higher than that of undoped NBT-6BT and NBT-9BT, respectively. Similar behavior is observed for $F_{d}$ and $F_{v}$, where the Zn-doping increases the FOM at room temperature, as seen in Figure 6e,f and Figure 7e,f. Zn-doping results in an increase in the temperature-independent behavior from 303–350 K for NBT-6BT to 303–380 K for NBT-6BT-0.001Zn. However, the FOMs after the tenth heating cycle showed a better temperature-independent behavior for Zn-doped NBT-6BT and NBT-9BT (Figure A1 and Figure A2). Table 1 presents the FOMs at T_d_ for different NBT-yBT-xZn (y = 6, 9 and x = 0, 0.005, 0.01) after the tenth heating cycle. Most studies focus on enhancing the pyroelectric properties at T_d_, while lowering it to near room temperature. However, for practical applications, wide temperature-independent properties are needed. These results show that NBT-yBT-xZn can be a promising candidate for pyroelectric applications in a wide temperature range.

## 4. Conclusions

In the present work, pyroelectric properties and relevant FOMs for Na_1/2_Bi_1/2_TiO_3_-yBaTiO_3_-xZn ceramics (y = 6, 9 and x = 0, 0.005, 0.01) were investigated. Zn-doping increases the depolarization temperature by almost ~40 K compared to pure NBT-6BT and NBT-9BT. Both relative permittivity and dielectric loss decrease with increasing ZnO content which is beneficial for the pyroelectric applications. Zn-doping increases the range of temperature-independent pyroelectric coefficient (*p*) behavior compared to undoped Na_1/2_Bi_1/2_TiO_3_-yBaTiO_3_ (y = 6, 9) and features higher *p* in comparison to other lead-free and PZT ceramics. Pyroelectric detector FOMs (F_d_) are enhanced with Zn doping to 8.16, 21.8 and 26 × 10^6^ Pa^−1/2^ for x = 0, 0.005 and 0.01 in NBT-6BT-xZn, respectively, at room temperature. These results demonstrate that NBT-6BT-xZn (x = 0.01) is a promising material for infrared detectors and other pyroelectric applications in a wide operating temperature range of ~80 K (300–380 K).

## Figures and Tables

**Table 1:** Comparison of the pyroelectric coefficient and FOMs (after the tenth cycle of heating) at room temperature and depolarization temperature (T_d_) for some important bulk materials.

Compositions	*p* × 10^−4^ (C m^−2^ K^−1^)	*F_i_* × 10^−10^ (C m J^−1^)	*F_v_* × 10^−3^ (m^2^ C^−1^)	*F_d_* × 10^−6^ (Pa^−1/2^)	*Fc* × 10^−4^ (C m^−2^K^−1^)	Ref.
**at room temperature**						
NBT-6BT	3.40	1.07	17.9	8.16	0.11	Present work
NBT-6BT-0.005Zn	2.18	1.41	28.20	21.8	0.16	Present work
NBT-6BT-0.01Zn	2.43	1.44	34.5	26.3	0.18	Present work
NBT-9BT	1.72	0.81	10	5.06	0.08	Present work
NBT-9BT-0.005Zn	1.71	0.83	13.6	11.3	0.09	Present work
NBT-9BT-0.01Zn	1.51	1.07	23	17.4	0.139	Present work
NBT-6BT	3.14	1.12	0.021	9.08	1.58	[24,26,27,28]
NBT-6Ba_1.02_T	3.54	1.244	0.95	8.3		[25]
N_0.52_B_0.52_T-6BT	6.99	2.50	0.047	16.63	2.86	[24]
NBT-6BT-0.002Ta	7.14	2.55	0.033	1.29	2.42	[27]
NBT-6BT-0.005La	7.42	2.65	0.048	1.4	2.98	[26]
0.005La-NBT-6BT-0.002Ta	12.92	4.61	0.078	2.76	2.57	[28]
BNT-BKT-BT	3.66	2.15	0.026	15.41		[43]
0.95NBT-0.05BT- Mn-doped *	8.34	1.11	0.016	8.64	0.29	[44]
BNKBT	3.25	1.95	0.026	13.43		[8]
KNLNTS	1.90	0.93	0.007	11.51		[8]
Ca_0.15_(Sr_0.5_Ba_0.5_)_0.85_Nb_2_O_6_	3.61	1.71	0.02			[45]
NBT-0.07BZT	5.71	2.03	0.022	10.50		[8]
PZT	4.14	1.42	0.008	9.01		[8]
BaSn_0.05_Ti_0.95_O_3_	5.57	3.55	0.018			[37]
Ba_0.85_Sr_0.15_TiO_3_	7.2	3.14	0.009			[46]
**at around depolarization temperature**						
NBT-6BT (352 K)	974	2.81	72.17	8.25	0.25	Present work
NBT-6BT-0.005Zn (395 K)	121	2.94	58.94	6.01	0.22	Present work
NBT-6BT-0.01Zn(400 K)	964	68.8	41126	4156	6.03	Present work
NBT-9BT(420 K)	605	2.75	41.7	3.79	0.19	Present work
NBT-9BT-0.005Zn(442 K)	187	4.05	116	10.25	0.32	Present work
NBT-9BT-0.01Zn(442 K)	108	5.77	312	27.36	0.52	Present work
N_0.50_B_0.49_T-6BT(393 K)	47.8	15.13	82.24	50.77	1.03	[30]
N_0.5_B_0.5_T-6BT(383 K)	87	28.19	90.52	66.44	1.46	[30]
N_0.5_B_0.51_T-6BT(353 K)	103	28.44	148.13	97.49	2.02	[30]
N_0.51_B_0.5_T-6BT (353 K)	105	34.37	223.75	120	2.41	[30]
NBT-6BT (363 K)	23.9	8.55	0.19	65.5		[24,26,27,28]
NBT-6BT (363 K)	53.3	19.7	0.14	75.6		[25]
NBT-6Ba_1.02_T (358 K)	741	260	1.64	915		[25]
N_0.52_B_0.52_T-0.06BT (355.7 K)	75.33	26.92	0.39	138.72		[24]
NBT-6BT-0.002Ta (351.8 K)	146.1	52.2	0.48	19.7		[27]
NBT-6BT-0.005La (346.5 K)	86.1	30.8	0.52	15.8		[26]
0.005La-NBT-6BT-0.002Ta (334.3 K)	58.62	20.94	0.18	7.60		[28]

* Single crystal.

## Data Availability

The data that support the findings of this study are available within this article.
